# Study of Biomolecular Interactions of Mitochondrial Proteins Related to Alzheimer’s Disease: Toward Multi-Interaction Biomolecular Processes

**DOI:** 10.3390/biom10091214

**Published:** 2020-08-21

**Authors:** Erika Hemmerová, Tomáš Špringer, Zdeňka Krištofiková, Jiří Homola

**Affiliations:** 1 Institute of Photonics and Electronics of the Czech Academy of Sciences, Chaberská 1014/57, 182 51 Prague, Czech Republic; hemmerova@ufe.cz (E.H.); springer@ufe.cz (T.Š.); 2 National Institute of Mental Health, Topolová 748, 250 67 Klecany, Czech Republic; zdenakristofikova@gmail.com

**Keywords:** 17β-hydroxysteroid dehydrogenase 10 (17β-HSD10), amyloid beta (Aβ), biomolecular interaction analysis, cyclophilin D (cypD), kinetic parameters, surface plasmon resonance (SPR)

## Abstract

Progressive mitochondrial dysfunction due to the accumulation of amyloid beta (Aβ) peptide within the mitochondrial matrix represents one of the key characteristics of Alzheimer’s disease (AD) and appears already in its early stages. Inside the mitochondria, Aβ interacts with a number of biomolecules, including cyclophilin D (cypD) and 17β-hydroxysteroid dehydrogenase type 10 (17β-HSD10), and affects their physiological functions. However, despite intensive ongoing research, the exact mechanisms through which Aβ impairs mitochondrial functions remain to be explained. In this work, we studied the interactions of Aβ with cypD and 17β-HSD10 in vitro using the surface plasmon resonance (SPR) method and determined the kinetic parameters (association and dissociation rates) of these interactions. This is the first work which determines all these parameters under the same conditions, thus, enabling direct comparison of relative affinities of Aβ to its mitochondrial binding partners. Moreover, we used the determined characteristics of the individual interactions to simulate the concurrent interactions of Aβ with cypD and 17β-HSD10 in different model situations associated with the progression of AD. This study not only advances the understanding of Aβ-induced processes in mitochondria during AD, but it also provides a new perspective on research into complex multi-interaction biomolecular processes in general.

## 1. Introduction

Alzheimer’s disease (AD) is the most widespread neurodegenerative disorder, which is characterized by decline of memory and cognitive functions due to extensive neuronal death. The main hallmarks found in the brains of AD patients are senile plaques (consisting of extracellular clusters of amyloid beta (Aβ) peptides) and neurofibrillary tangles (consisting of intracellular deposits of hyperphosphorylated protein tau). Several hypotheses explaining the role of Aβ and protein tau in the progression of AD have been proposed; however, despite years of intensive research, the causes and pathogenic mechanisms of AD are still not fully understood [1].

A strong body of evidence suggests that functions of neuronal synaptic mitochondria deteriorate in the early stages of AD [2,3], in which Aβ may be significantly involved [4,5]. Aβ is produced from amyloid precursor protein in the form of fragments of different lengths [6,7]. Aβ consisting of 40 and 42 amino acids (Aβ_1–40_ and Aβ_1–42_) are the most common physiological forms of Aβ and represent 80–90% and 5–10% of the total Aβ secreted, respectively [8]. It has been established that during AD, degradation mechanisms of Aβ are impaired [9], and production of Aβ is increased [10] and skewed towards Aβ_1–42_ [11]. The different fragments of Aβ exhibit different oligomerization dispositions, with Aβ_1-42_ being more prone to form oligomers than Aβ_1–40_ [12,13]. Such oligomerization is known to increase neuronal toxicity of Aβ [14]. In early stages of AD, Aβ starts to accumulate inside the mitochondrial matrix [5,15], where it interacts with a broad range of mitochondrial biomolecules [16,17,18]. The major Aβ binding partners associated with AD are cyclophilin D (cypD) and 17β-hydroxysteroid dehydrogenase 10 (17β-HSD10). These interactions have been shown to lead to mitochondrial dysfunctions [3,19], including impaired energy metabolism [20], production of reactive oxygen species (ROS) [21], perturbation in calcium homeostasis [22], and formation and opening of the mitochondrial permeability transition pores (mPTPs) [23]. However, the exact mechanisms behind these processes remain largely unknown.

The interactions between Aβ and cypD or 17β-HSD10 have been studied by several methods, including ELISA, crystallography, surface plasmon resonance (SPR), or co-immunoprecipitation; however, only a few studies have focused on the kinetic aspects of the interactions so far. Using radioactive and non-radioactive (ELISA) ligand binding assays, Yan et al. studied the equilibrium properties of the interaction between 17β-HSD10 and Aβ, and determined equilibrium dissociation constants (K_D_) of 42–88 nM for both fragments of Aβ (Aβ_1–40_ and Aβ_1–42_) [24,25]. Similar results were obtained by Lustbader et al. by the radioactive and fluorescence ligand binding assays, who reported K_D_ of 38.4 ± 4.6 nM and 55.8 ± 10.9 nM for the interaction of 17β-HSD10 with Aβ_1–40_ and Aβ_1–42_, respectively [26]. However, neither study paid attention to the oligomerization state of Aβ, which is an important factor that may have a substantial effect on the interaction. The equilibrium properties of the interaction between cypD and Aβ (Aβ_1–40_ and Aβ_1–42_) were investigated by Du et al. [27]. They considered different oligomerization dispositions of different Aβ fragments and determined K_D_ values for the interactions of cypD with monomeric Aβ_1–40_ and Aβ_1–42_ as well as with oligomeric Aβ_1–40_ and Aβ_1–42_ to be 1.7 μM, 164 nM, 227 nM, and 4 nM, respectively. However, it should be noted that in these experiments, the biomolecules were dissolved in water in the absence of ions. As demonstrated by our recent work concerning the interaction between cypD and 17β-HSD10 [28], the binding between mitochondrial proteins is sensitive to the properties of the medium in which the interactions take place (pH, concentration of ions). This suggests that the interaction parameters determined under non-physiological conditions should be used with caution. Investigation of kinetic parameters of the interactions of Aβ and 17β-HSD10 has also been attempted in order to provide insight into the molecular interaction dynamics. Yan et al. studied the interaction between Aβ_1–40_ and 17β-HSD10, and determined the interaction kinetic parameters (k_a_, k_d_, and K_D_) [29]. In their work, the interaction was observed only for the oligomeric form of Aβ_1–40_, while no interaction was observed for the monomeric form, suggesting that 17β-HSD10 can bind Aβ_1–40_ only when it is in its oligomeric form. Based on these studies, it can be concluded that the current knowledge of the interactions between Aβ and cypD or 17β-HSD10 is hampered by two major issues: uncertainty about the oligomerization state of the interacting Aβ and different experimental conditions (often far from physiological) used in different studies. Therefore, systematic study of the interactions between different fragments of Aβ and cypD and 17β-HSD10 under identical and physiologically relevant conditions is essential.

In this work, we study, for the first time, the interactions of Aβ (Aβ_1–40_ and Aβ_1–42_) with cypD and 17β-HSD10 under conditions relevant to the environment of mitochondrial matrix (relevant pH and levels of ions), but in the absence of all the other interfering biomolecules to establish the kinetic parameters (k_a_, k_d_, K_D_) of these interactions and to enable direct comparison of the affinities of Aβ towards cypD and 17β-HSD10. In addition, we show how the knowledge of kinetic parameters of individual interactions may contribute to the description of the complex interplay of biomolecular interactions in mitochondria. We present a model describing the parallel interactions between cypD, 17β-HSD10, and different fragments of Aβ in different oligomerization states, and show that the model allows estimation of the evolution of levels of free biomolecules and their complexes under selected conditions associated with the progression of AD.

## 2. Materials and Methods

### 2.1. Reagents

NaCl, NaOH, KCl, MgCl_2_, hexafluoroisopropanol (HFIP), NH_4_OH, bovine serum albumin (BSA), sinapinic acid (SA), acetonitrile (ACN), trifluoroacetic acid (TFA), and all buffers: sodium acetate (SA10; 10 mM, pH 5.0), MES (10 mM, pH 5.0), HEPES, phosphate-buffered saline (PBS; 10 mM phosphate, 2.9 mM KCl, 140 mM NaCl, pH 7.4), and high ionic strength PBS (PBS_Na_; 10 mM phosphate, 2.9 mM KCl, 750 mM NaCl, pH 7.4) were purchased from Sigma-Aldrich, Czech Republic. Oligo-ethylene glycol thiols 11-mercapto-hexa(ethyleneglycol)undecyloxy acetic acid (HS-C_11_-(EG)_6_-OCH_2_-COOH) and 11-Mercapto-tetra(ethyleneglycol)undecanol (HS-C_11_-(EG)_4_-OH) were purchased from Prochimia, Poland. Ethanolamine hydrochloride (EA), N-hydroxysuccinimide (NHS), and 1-ethyl-3-(3-dimethylaminopropyl)-carbodiimide hydrochloride (EDC) were purchased from Biacore, Sweden. All buffers were prepared using deionized Milli-Q water (Merck, Czech Republic). Human recombinant 17β-HSD10 (NCBI Gene ID: 3028), human recombinant cypD (NCBI Gene ID: 10105), and an antibody against cypD (Ab(cypD)) were purchased from Fitzgerald, USA. In addition, 17β-HSD10 (purified using the procedure by Aitken et al. [30]) with verified catalytic activity was prepared and kindly provided by the research group of prof. Musílek (the University of Hradec Králové, Czech Republic). An antibody against 17β-HSD10 (Ab(17β-HSD10)) was purchased from Biolegend, USA. Aβ (human, synthetic), i.e., Aβ_1–40_ (PDB: 1AML) and Aβ_1–42_ (PDB: 1IYT) were obtained from AnaSpec, USA, dissolved in 1% NH_4_OH and diluted by PBS to obtain the stock concentration of 100 μM. The running buffer RB1 (and RB2) was prepared as 10 mM HEPES in Milli-Q with addition of BSA (200 μg/mL). The pH was adjusted by NaOH to 7.4 and NaCl, KCl, and MgCl_2_ were used to adjust the concentration of Na^+^, K^+^, and Mg^2+^ to 5, 140, and 1 mM (or 5 mM for RB2), respectively.

### 2.2. Surface Plasmon Resonance (SPR) Biosensor

We used a six-channel SPR biosensor platform based on the wavelength spectroscopy of surface plasmons (Plasmon VI) developed at the Institute of Photonics and Electronics, Prague. In this SPR platform, the angle of incidence of the light beam is fixed and changes in the resonance wavelength of surface plasmons are measured by analyzing the spectrum of polychromatic light reflected from an SPR chip. The resonance wavelength is sensitive to changes in the refractive index caused by the binding of biomolecules to the surface of an SPR chip. A shift in the resonance wavelength of 1 nm represents a change in the protein surface coverage of 17 ng/cm^2^. The SPR chips used in this study were prepared by coating microscope glass slides obtained from Marienfeld, Germany with thin layers of titanium (1–2 nm) and gold (48 nm) via e-beam evaporation in vacuum. The SPR platform was combined with a dispersionless microfluidic module [31]. The active temperature stabilization unit allowed maintaining of temperature within the system with a precision of 0.01 °C. The experiments reported in this study were performed at a temperature of 25 °C and a flow rate of 20 µL/min.

Prior to the experiments, the surface of an SPR chip was modified by a self-assembled monolayer of mixed thiols, on which specific antibodies, Ab(cypD) or Ab(17β-HSD10), were immobilized using the amino-coupling method as described previously [32]. Briefly, a clean SPR chip was immersed in a 3:7 molar mixture of HS-C_11_-(EG)_6_-OCH_2_-COOH and HS-C_11_-(EG)_4_-OH (ethanol solution, total concentration of 0.2 mM), then, incubated in the dark for 10 min at 40 °C, and then, for at least 12 h at a room temperature. Before the chip was mounted in the SPR platform, it was rinsed with ethanol and Milli-Q water, and then, dried with a stream of nitrogen. First, the mixture of 12.5 mM NHS and 62.5 mM EDC (in Milli-Q water) was injected (10 min) to activate carboxylic groups. Then, Ab(cypD) or Ab(17β-HSD10) at a concentration of 10 μg/mL in SA10 was pumped through the flow-cell until the response to the immobilized antibody levelled off (~15 min). Then, PBS_Na_ was applied (5 min) to remove the non-covalently attached antibody. Finally, 500 mM EA was injected (5 min) to deactivate the unreacted carboxylic groups. The SA10 running buffer was exchanged for MES and then, detection channels were exposed to 100 nM cypD or 100 nM 17β-HSD10 in MES until the particular sensor response was reached, while the reference channels were kept in MES. Then, all the channels were washed by MES for at least 20 min. In the procedure of cypD immobilization, the detection and reference channels were consequently exposed to PBS_Na_ (5 min) in order to prevent uncontrolled dissociation of cypD from the surface.

### 2.3. Preparation of Aβ

Aβ used in our study was prepared by three different procedures. As Aβ exhibits a high tendency to form oligomers, we assumed that Aβ in the stock solution occurred in the oligomeric form and the oligomeric Aβ sample was prepared by a simple dilution in the running buffer (Preparation A). In order to produce Aβ samples with an Aβ state as close to monomeric as possible, we used NaOH [33] (Preparation B) and HFIP [34] (Preparation C), the agents that were previously demonstrated to disassemble Aβ oligomers into monomers. In Preparation A, Aβ stock solution was diluted by the running buffer to obtain the particular concentration of Aβ. In Preparation B, Aβ stock solution was diluted by 12.5 mM NaOH in the volume ratio of 1:4 and sonicated for 5 min. Then, the sample was diluted by the running buffer to obtain the particular concentration of Aβ. In Preparation C, Aβ from stock solution was mixed with HFIP in the volume ratio of 1:9 and vortexed for 1 min. Then, the solvent was evaporated by a stream of nitrogen and solid Aβ was dissolved in the running buffer to obtain the particular concentration of Aβ. The prepared samples were immediately injected into the flow-cell of the SPR biosensor.

### 2.4. Characterization of Oligomerization State of Aβ

The oligomerization state of Aβ used in our experiments was analyzed by the matrix assisted laser desorption/ionization time-of-flight (MALDI-TOF) and SPR methods. Two types of samples of Aβ_1–42_ were prepared: Sample 1 containing freshly dissolved Aβ_1–42_ and Sample 2 containing freshly dissolved Aβ_1–42_ after 5 days of incubation at 37 °C. Both samples were prepared by Preparations A–C described above.

MALDI-TOF analysis was performed using UltrafleXtremeTM MALDI TOF/TOF mass spectrometer (Bruker Daltonics, Germany) with 1 kHz smartbeam II laser. The measurements were realized in positive linear mode, with the mass range of 5–100 kDa. The accelerating voltage was set at 25 kV. Spectra were obtained by accumulating of 10,000 shots. Samples of Aβ_1–42_ were prepared for the analysis by dilution with 10 mM HEPES, pH 7.4, to obtain the final concentration of 4.5 μM. An amount of 30 mg/mL SA in 50% ACN containing 0.1% (v/v) TFA was used as a matrix solution.

We further evaluated binding of Aβ_1–42_ in Samples 1 and 2 prepared by Preparation A to cypD using SPR biosensor. The level of the cypD immobilized on the sensor surface of the detection channel in these experiments was, when expressed in terms of sensor response, about 2 nm. Then, RB2 was pumped through the flow-cell until the stable baseline was obtained and Samples 1 and 2 were simultaneously injected into the detection and reference channels and flowed along the sensor surface for 5 min. Then, RB2 was injected again. Finally, the sensor response obtained in the reference channel was subtracted from that obtained in the detection channel.

Analogously, the binding of Aβ (both Aβ_1–40_ and Aβ_1–42_ prepared by Preparations A–C) to cypD immobilized on the surface of SPR biosensor was evaluated.

### 2.5. Characterization of cypD and 17β-HSD10

The biological activity of cypD used in our experiments was evaluated by the vendor and specified as >120 nM/min/μg (the amount of enzyme that cleaves 1 μM of suc-AAFP-pNA per minute at 1 °C in Tris-Hcl pH 8.0 using chymotrypsin). In order to explore the integrity and activity of the used 17β-HSD10, we compared 17β-HSD10 used in our experiments (17β-HSD10_commercial_) to 17β-HSD10 with verified catalytic activity (17β-HSD10_UHK_) provided by the University of Hradec Králové. In this experiment, both 17β-HSD10_commercial_ and 17β-HSD10_UHK_ were immobilized on the SPR chip via Ab(17β-HSD10) and the interaction between Aβ_1–40_ (prepared by Preparation C) and Aβ_1–42_ (prepared by Preparation A) and the immobilized 17β-HSD10 was characterized by the SPR method. As follows from Figure S4 in Supplementary information, the original 17β-HSD10 and the newly obtained one exhibited comparable affinities to both Aβ_1–40_ and Aβ_1–42_. Therefore, we believe that both proteins, cypD and 17β-HSD10, used in our study, are suitable and relevant for the investigation of mitochondrial processes.

### 2.6. Determination of Kinetic Parameters

In the biomolecular interaction analysis experiments, RB1 was flowed along the SPR chip functionalized with cypD (surface coverage corresponding to the sensor response of 1 nm) or with 17β-HSD10 (surface coverage corresponding to the sensor response of 1.5 nm) until the stable baseline was reached. Aβ was prepared using the procedures described in the section “Preparation of Aβ“, in particular, monomeric forms of Aβ_1–40_ and Aβ_1–42_ were prepared using Preparation C and Preparation B, respectively, while an oligomeric form of Aβ_1–42_ was prepared by Preparation A. A series of five concentrations of each form of Aβ was prepared: 1.1, 0.66, 0.44, 0.22, 0.11 μM; 1.1, 0.55, 0.22, 0.11, 0.055 μM; 2.2, 1.7, 1.1, 0.55, 0.22 μM; and 4.4, 1.7, 1.1, 0.55, 0.22 μM for monitoring of the binding of monomeric Aβ_1–42_ to cypD; oligomeric Aβ_1–42_ to cypD as well as oligomeric Aβ_1–42_ to 17β-HSD10; monomeric Aβ_1–40_ to cypD as well as monomeric Aβ_1–42_ to 17β-HSD10; and monomeric Aβ_1–40_ to 17β-HSD10, respectively. The Aβ samples were injected into both detection and reference channels for 10 min to monitor the association phase. Then, the surface was exposed to RB1 for 30 min, to monitor the dissociation phase. The reference-compensated binding curves (sensor responses from the reference channels were subtracted from those obtained in the particular detection channels) were globally fitted using the BIAevaluation software version 4.1 from Biacore, Sweden and 1:1 Langmuir model. The value of the maximal binding capacity was determined by local fitting due to the variations in protein surface coverage among different channels. The final kinetic parameters were calculated as a mean of kinetic parameters determined by the least square fitting of at least three independent sensorgrams.

## 3. Results and Discussion

### 3.1. Characterization of Oligomerization State of Aβ

The control over the oligomerization state of Aβ and its characterization is an important but non-trivial task for which there is currently no generally accepted approach available. Recently, several methods have been proposed to characterize the oligomerization state of Aβ, including mass spectrometry, electron microscopy [35], fluorescence, NMR [34], electrophoresis, chromatography or light scattering [36], or SPR [37], and several agents such as NaOH [33] and HFIP [34] have been demonstrated to disassemble Aβ oligomers into monomers.

In order to determine the oligomerization state of Aβ used in our experiments, we performed the MALDI-TOF analysis of different samples of Aβ_1–42_. Specifically, two types of samples of Aβ_1–42_, which were expected to represent different initial oligomerization states (Samples 1 and 2), were prepared by Preparations A–C (see “Preparation of Aβ” and “Characterization of the oligomerization state of Aβ” sections in Materials and Methods). The obtained mass spectra are shown in Figure S1 in Supplementary information. They suggest that both Aβ monomer and low oligomers were present in all of the analyzed samples. These results contradict previously published studies; for instance, under the conditions used for the preparation of Sample 2 (high concentration—100 μM, high temperature—37 °C, and long incubation time—5 days), Aβ_1–42_ was demonstrated to oligomerize by Garai et al. [13].

In order to address this discrepancy, we performed additional SPR experiments, in which we evaluated the binding of Aβ_1–42_ in Samples 1 and 2 prepared by Preparation A to cypD immobilized on the sensor surface. The obtained sensorgrams are shown in Figure S2 in Supplementary information. We observed significantly lower sensor response for Sample 1 than for Sample 2. In the SPR method, the sensor response is given by the mass of the analyte and its affinity to the immobilized capture molecule. As oligomers have been reported to exhibit higher affinity to cypD than monomers [27], lower sensor response to the binding of Aβ to cypD corresponds to a lower oligomerization state of Aβ. Therefore, we conclude that the analyzed Aβ samples (Sample 1 and Sample 2) indeed contained Aβ in different oligomerization states. We believe that contradictory outcomes of SPR and MALDI-TOF experiments may be caused by the laser-induced disassembly of oligomers during the ionization step in the MALDI-TOF experiments. This indicates that the MALDI-TOF method (at least under given experimental conditions) was not able to provide reliable information on the oligomerization state of Aβ in our samples.

In addition, in order to demonstrate the effect of different preparation procedures, we measured the SPR sensor responses to the binding of Aβ (Aβ_1–40_ and Aβ_1–42_ prepared by Preparations A–C) to cypD immobilized on the sensor surface. The typical sensor responses obtained for different Aβ samples are shown in Figure S3 in Supplementary information. Our results demonstrate that both Aβ_1–40_ and Aβ_1–42_ exposed to NaOH or HFIP exhibit lower oligomerization states than Aβ in the stock solution.

Although the MALDI-TOF experiments did not provide an answer on the level of oligomerization of Aβ, we relied on the previously published works using NaOH and HFIP to disassemble the oligomers of Aβ [33,34] and on our results obtained by the SPR biosensor, demonstrating that these agents indeed reduce the oligomerization state of Aβ. Therefore, in the following text, we will assume that Aβ prepared by Preparation A represents the oligomeric form, while Aβ_1–42_ prepared by Preparation B and Aβ_1–40_ prepared by Preparation C represent the forms which are close to monomeric (might contain the mixture of monomers and low oligomers) and we will refer to it as to the monomeric state.

### 3.2. Determination of Kinetic Parameters of the Interactions between Aβ and cypD or 17β-HSD10

In this study, we investigated kinetic aspects of the interactions between Aβ (Aβ_1–40_ and Aβ_1–42_) and cypD or 17β-HSD10, under the conditions which simulate the physiological environment in the mitochondrial matrix (in the presence of 140 mM K^+^ [38,39] and 1 mM Mg^2+^ [40,41]), but in the absence of all other biomolecules. In the first stage of experiments, we worked with Aβ_1–40_ and Aβ_1–42_ fragments in the monomeric form; then, interactions involving Aβ_1–40_ and Aβ_1–42_ oligomers were studied.

**Figure 1:**
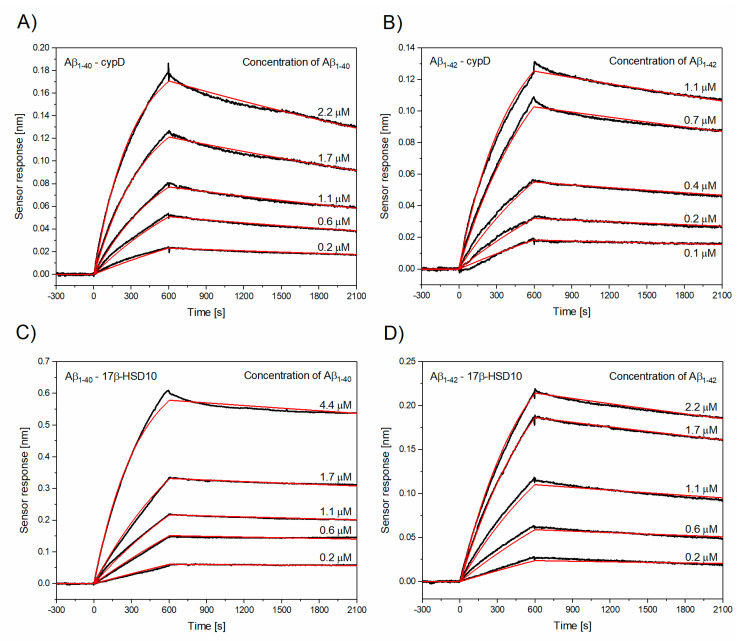
Reference-compensated sensorgrams (black lines) and their global fits (red lines) obtained for the set of five different concentrations of monomeric Aβ_1–40_/Aβ_1–42_ binding to cypD or to 17β-HSD10, respectively. (**A**) Binding of monomeric Aβ_1–40_ to cypD, (**B**) binding of monomeric Aβ_1–42_ to cypD, (**C**) binding of monomeric Aβ_1–40_ to 17β-HSD10, (**D**) binding of monomeric Aβ_1–42_ to 17β-HSD10.

Figure 1 shows the reference-compensated sensorgrams obtained using a multichannel SPR biosensor (see “Surface plasmon resonance (SPR) biosensor” section in Materials and Methods). It presents a temporal sensor response to the interaction between cypD or 17β-HSD10 immobilized on the surface of the sensor and monomeric Aβ (Aβ_1–40_ or Aβ_1–42_) for five different concentrations of Aβ. The kinetic parameters (k_a_, k_d_, and K_D_) obtained by global fitting of the sensorgrams are listed in Table 1. The highest k_a_ was obtained for the binding of Aβ_1–42_ to cypD; k_a_ of the binding of Aβ_1–40_ to cypD was lower by a factor of two and k_a_ of the binding of Aβ_1–40_ to 17β-HSD10 was comparable with that of the binding of Aβ_1–42_ to 17β-HSD10 and lower by a factor of four in comparison with that of Aβ_1–42_–cypD. The interaction between Aβ and cypD and the interaction between Aβ_1–42_ and 17β-HSD10 were found to exhibit similar k_d_ values; k_d_ obtained for the interaction between Aβ_1–40_ and 17β-HSD10 was lower by a factor of three, indicating high stability of the Aβ_1–40_/17β-HSD10 complex. The corresponding K_D_ values suggest that the interaction between Aβ_1–42_ and cypD and the interaction between Aβ_1–40_ and 17β-HSD10 exhibit the highest affinity (lowest K_D_), while Aβ_1–40_ binds cypD with affinity lower by a factor of two and 17β-HSD10 with affinity higher by a factor of three than Aβ_1–42_ does.

**Figure 2:**
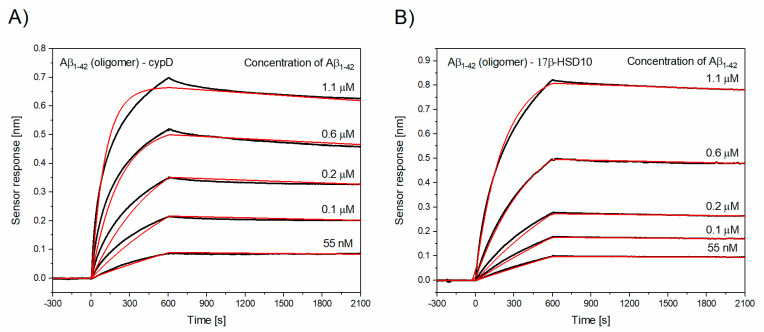
Binding of oligomeric Aβ_1–42_ to cypD or to 17β-HSD10. Reference-compensated sensorgrams (black lines) and their global fits (red lines) obtained for the set of five different concentrations of oligomeric Aβ_1–42_ binding to (**A**) cypD and (**B**) to 17β-HSD10. (**A**) Binding of oligomeric Aβ_1–42_ to cypD, (**B**) binding of oligomeric Aβ_1–42_ to 17β-HSD10.

In order to simulate the conditions occurring during the progression of AD, we also investigated interactions involving oligomeric Aβ_1–42_. Figure 2 shows the temporal sensor response to the interaction between cypD or 17β-HSD10 molecules immobilized on the sensor and oligomeric Aβ_1–42_. As follows from Table 2, the oligomeric Aβ_1–42_ exhibits a significantly lower K_D_ for the interactions with both cypD and 17β-HSD10, indicating that the affinity of oligomeric Aβ_1–42_ exceeds that of the monomeric Aβ_1–42_. The observed decrease in K_D_ is caused by both the increased k_a_ and decreased k_d_. This suggests that the complexes of the oligomeric Aβ_1–42_ and cypD or 17β-HSD10 are formed faster and are more stable than the complexes of the monomeric Aβ. However, it is worth noting that analytes that tend to form multimers might show abnormally slow binding, as a result of the effective decrease in their molar concentration due to the multimer formation [42] and therefore, the affinity of Aβ towards cypD and 17β-HSD10 might be even higher than reported here.

Our results confirm that: (i) Aβ_1–42_ exhibits higher affinity towards cypD than Aβ_1–40_ and (ii) oligomeric Aβ_1–42_ has higher affinity towards cypD than the monomeric, as suggested by Du et al. [27]. However, the K_D_ values obtained in our study are considerably lower (by a factor of about ten for the interaction between cypD and Aβ_1–40_ and by a factor of about three for interaction between cypD and Aβ_1–40_). These differences may arise from different experimental conditions used to study the interactions and qualitatively agree with the results of our recent study showing the lower binding efficiency between mitochondrial proteins (cypD and 17β-HSD10) in the absence of ions [28]. The K_D_ value for the interaction between Aβ and 17β-HSD10 obtained in our study agrees well with the previously reported values [24,25,29], which were obtained in tris, phosphate, or carbonate buffers. However, our results differ qualitatively from those reported by Yan et al. [29]. In contrast to the work of Yan et al. [29], we observed the binding of both monomeric and oligomeric fragments of Aβ to 17β-HSD10.

### 3.3. Modeling the Complex Biomolecular Interaction Interplay in Mitochondria

Current biosensor-based biomolecular interaction analysis tends to focus on individual biomolecular interactions, identification of the interaction models, and determination of kinetic parameters of these models. The kinetic parameters k_a_ and k_d_ describe how quickly the interacting partners form complexes and how quickly such complexes dissociate.

Herein, we show how the results of individual interaction studies can be extended to address a more complex problem—to describe the system with multiple concurrent biomolecular interactions. We introduce a model of the biomolecular interactions of Aβ, cypD, and 17β-HSD10 using the k_a_ and k_d_ parameters determined above. The model assumes a set of four parallel interactions between Aβ_1–40_ or Aβ_1–42_ and cypD or 17β-HSD10 that can be described by Equation (1). The corresponding differential equations expressing the particular reaction rates are given in Supplementary information. 

The system of biomolecular interactions studied in the developed model Equation (1).

(1) $$\begin{array}{c} A\beta_{1-40}+cypD⇋{\;}_{k_{d1}}^{k_{a1}}\;A\beta_{1-40}/cypD\\ A\beta_{1-42}+cypD⇋{\;}_{k_{d2}}^{k_{a2}}\;A\beta_{1-42}/cypD\\ A\beta_{1-40}+17\beta -HSD10⇋{\;}_{k_{d3}}^{k_{a3}}\;A\beta_{1-40}/17\beta -HSD10\\ A\beta_{1-42}+17\beta -HSD10⇋{\;}_{k_{d4}}^{k_{a4}}\;A\beta_{1-42}/17\beta -HSD10 \end{array}$$

We applied the developed model to analyze four different model situations: (A) Physiological state—establishment of equilibrium in the presence of small concentrations of Aβ with a Aβ_1–40_:Aβ_1–42_ ratio of 9:1 corresponding to physiological state [8]; (B) AD1—the equilibrium established in situation (A) is disturbed by imbalanced production of Aβ_1–40_ and Aβ_1–42_ (the Aβ_1–40_:Aβ_1–42_ ratio changes to 1:1), while the overall concentration of the produced Aβ remains constant; (C) AD2—the equilibrium established in situation (A) is disturbed by imbalanced production of Aβ_1-40_ and Aβ_1–42_ (the Aβ_1–40_:Aβ_1–42_ ratio changes to 1:1) and by increased concentration of Aβ (by a factor of 100); and (D) AD3—the equilibrium established in situation (A) is disturbed by imbalanced production of Aβ_1–40_ and Aβ_1–42_ (the Aβ_1–40_:Aβ_1–42_ ratio changes to 1:1), by increased concentration of Aβ (by a factor of 100), and by the oligomerization of Aβ_1-42_.

**Figure 3:**
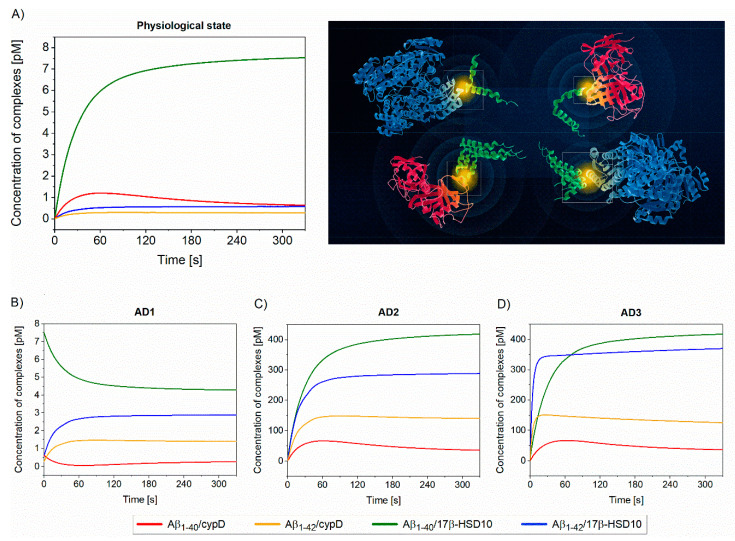
Simulation of the concurrent interactions between Aβ, cypD, and 17β-HSD10. (**A**) Simulation of the physiological state, (**B**–**D**) simulation of multiple situations associated with the onset of AD. The particular parameters for (**A**–**D**) are listed in Table S1.

As the absolute concentrations of Aβ, cypD and 17β-HSD10 in mitochondria are still a subject of research, in our simulations, we assumed a ratio of cypD:17β-HSD10 of 1:7 [43] and much smaller initial concentrations of Aβ in comparison to cypD and 17β-HSD10. The particular parameters used in the model are summarized in Table S1 in Supplementary information. The output of the model, expressed in terms of concentrations of particular complexes as a function of time, is depicted in Figure 3.

Figure 3A describes the time evolution of the interactions (determined using Equation (1)), when the interacting biomolecules, Aβ_1–40_, Aβ_1–42_, cypD, and 17β-HSD10 present at their physiological concentrations, are brought into contact. This suggests that the level of Aβ_1–40_/17β-HSD10 complex significantly exceeds those of other complexes, even though k_a_ of the interaction of Aβ_1–40_–17β-HSD10 is lower than k_a_ of all the other considered interactions (suggesting slower complex formation). This is due to (i) low k_d_ that makes Aβ_1–40_/17β-HSD10 more stable and (ii) significantly higher concentrations of 17β-HSD10 and Aβ_1–40_ in the system in comparison with cypD and Aβ_1–42_. Interestingly, as the interaction between Aβ_1–42_ and cypD exhibits high k_a_ and high k_d_ (indicating fast formation of less stable complex), the level of Aβ_1–40_/cypD initially increases. However, due to a high rate of dissociation, Aβ_1–40_ is gradually consumed by 17β-HSD10 and forms the more stable Aβ_1–40_/17β-HSD10. Therefore, the level of Aβ_1–40_/cypD subsequently decreases. The model implies that, under physiological conditions, 17β-HSD10 is a dominant binding partner for Aβ present in the mitochondrial matrix. Figure 3B illustrates the changes in the levels of complexes when the physiological state is disturbed by imbalanced production of Aβ fragments favoring Aβ_1–42_. The model predicts a decrease in Aβ_1–40_/17β-HSD10 and Aβ_1–40_/cypD levels (due to the drop in Aβ_1–40_ concentration) and an increase in Aβ_1–42_/17β-HSD10 and Aβ_1–42_/cypD levels (due to increased concentration of Aβ_1–42_). This indicates that, even under the increased production of Aβ_1–42_, 17β-HSD10 remains the major binding partner of Aβ; however, due to the high affinity of Aβ_1–42_ to cypD (high k_a_, low k_d_), a significant amount of Aβ is also captured in the complex with cypD. Figure 3C shows the disturbance of the physiological state by increased production of overall Aβ in addition to the imbalanced production of Aβ fragments favoriting Aβ_1–42_. This situation results in significant increase of all the complexes due to a higher level of Aβ available for the binding; the levels of complexes are increased by about the same factor in comparison with AD2. Figure 3D represents the situation combining both the aspects of imbalanced and increased production of Aβ with oligomerization of Aβ_1–42_. As oligomeric Aβ_1–42_ exhibits higher k_a_ and lower k_d_ than its monomeric counterpart, the formation of complexes of oligomeric Aβ_1–42_ is faster and the complexes are more stable than those of monomeric Aβ_1–42_. The equilibrium level of Aβ_1–40_/17β-HSD10 is comparable to the level of Aβ_1–42_/17β-HSD10 and the level of total Aβ captured by 17β-HSD10 is higher by a factor of five than the level of Aβ captured by cypD.

The presented model considers the biomolecular interactions of Aβ, cypD, and 17β-HSD10 in their physiological environment (relevant pH and levels of ions), but in the absence of all the other biomolecular processes taking place in the mitochondrial matrix. This may be further expanded by including interactions with other important mitochondrial biomolecules in order to provide a more comprehensive picture of molecular processes taking place in mitochondria during physiological conditions as well as during AD.

## 4. Conclusions

In this study, we characterize the interactions between two fragments of Aβ (Aβ_1–40_, Aβ_1–42_) and two proteins of the mitochondrial matrix (cypD, 17β-HSD10) using the SPR biosensor method. In addition, we present a multi-interaction model that simulates concurrent interactions of Aβ with cypD and 17β-HSD10 and show the results of simulations for a variety of conditions (physiological and pathological). The multi-interaction model suggests that the favored production of Aβ_1–42_ over Aβ_1–40_, accumulation of Aβ, and oligomerization of Aβ_1–42_ occurring in AD have profound impact on the interactions between mitochondrial biomolecules and substantially influence the dynamics and equilibrium of the interactions in the mitochondrial matrix. We believe that this work represents the first step towards development of more comprehensive models that will incorporate the effects of other biomolecules in the mitochondrial matrix, which will further advance our understanding of physiological as well as AD-related processes.

## Figures and Tables

**Table 1:** Kinetic parameters of the interactions between monomeric Aβ (Aβ_1–40_ or Aβ_1–42_) and cypD or 17β-HSD10.

The Interaction	k_a_ [M^−1^s^−1^]	k_d_ [s^−1^]	K_D_ [nM]
Aβ_1–40_–cypD	(1.17 ± 0.15) × 10^3^	(1.74 ± 0.57) × 10^−4^	160.2 ± 57.8
Aβ_1–42_–cypD	(2.69 ± 1.22) × 10^3^	(1.39 ± 0.41) × 10^−4^	56.5 ± 5.4
Aβ_1–40_–17β-HSD10	(0.63 ± 0.05) × 10^3^	(0.47 ± 0.04) × 10^−4^	74.5 ± 1.8
Aβ_1–42_–17β-HSD10	(0.65 ± 0.25) × 10^3^	(1.12 ± 0.52) × 10^−4^	181.4 ± 16.0

**Table 2:** Kinetic parameters of the interactions between oligomeric Aβ_1–42_ and cypD or 17β-HSD10.

The Interaction	k_a_ [M^−1^s^−1^]	k_d_ [s^−1^]	K_D_ [nM]
Oligomeric Aβ_1–42_–cypD	(11.12 ± 1.09) × 10^3^	(0.61 ± 0.12) × 10^−4^	5.3 ± 1.2
Oligomeric Aβ_1–42_–17β-HSD10	(4.04 ± 0.62) × 10^3^	(0.31 ± 0.09) × 10^−4^	8.0 ± 3.7

## Data Availability

The protein interactions from this publication have been submitted to the IMEx (http://www.imexconsortium.org) consortium and assigned the identifier IM-28055.

## Supplementary Materials

The following are available online at https://www.mdpi.com/2218-273X/10/9/1214/s1, Figure S1: Spectra obtained from MALDI-TOF analysis, Figure S2: Sensor responses to the binding of Aβ_1–42_ to the immobilized cypD. Figure S3: The effect of preparation of Aβ on its the binding to cypD; Figure S4: Comparison of the binding properties of commercially available 17β-HSD10 (17β-HSD10_commercial_) and 17β-HSD10 with verified catalytic activity (17β-HSD10_UHK_), detail model description; Table S1: Parameters of the multi-interaction model for interactions between Aβ, cypD and 17β-HSD10 under selected conditions associated with the progression of AD.
