# Supplementary material for: Study of Biomolecular Interactions of Mitochondrial Proteins Related to Alzheimer’s Disease: Toward Multi-Interaction Biomolecular Processes
Source: Biomolecules. 2020 Aug 21;10(9):1214. doi: 10.3390/biom10091214 (PMC7563123; doi:10.3390/biom10091214)
Supplement: Supplementary file 1 [file biomolecules-10-01214-s001.pdf]

Study of biomolecular interactions of mitochondrial proteins related to Alzheimer's disease: toward multi-interaction biomolecular processes

Erika Hemmerová, Tomáš Špringer, Zdenka Krištofíková, Jiří Homola

SUPPLEMENTARY INFORMATION

Characterization of the oligomerization state of Aβ using MALDI-TOF and SPR biosensor

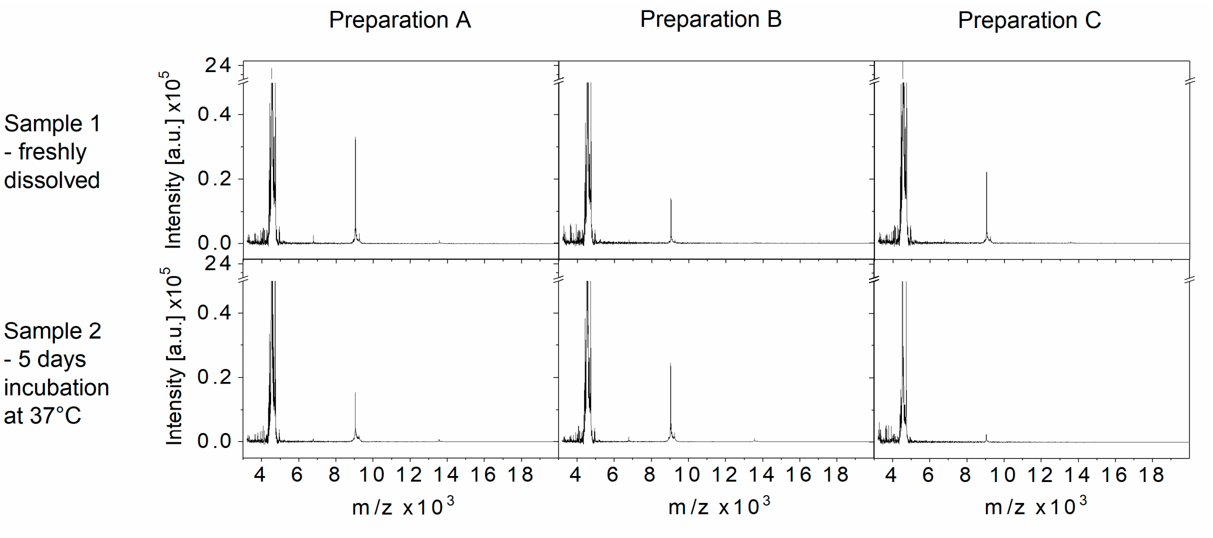

Figure S1: Spectra obtained from MALDI-TOF analysis of two different samples of $A\beta_{1-42}$ (freshly dissolved and after 5 days of incubation at 37°C) prepared by Preparations A-C.

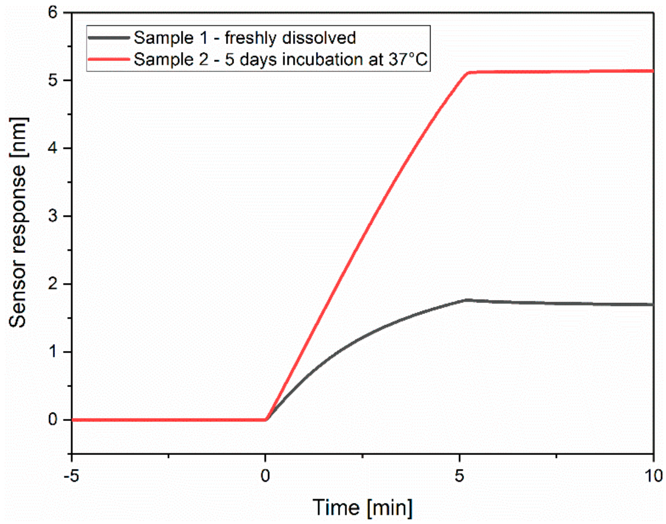

Figure S2: Sensor responses to the binding of $A\beta_{1-42}$ (Samples 1 and 2 prepared by Preparation A) to the immobilized cypD.

The effect of Preparations A-C on the oligomerization state of Aβ

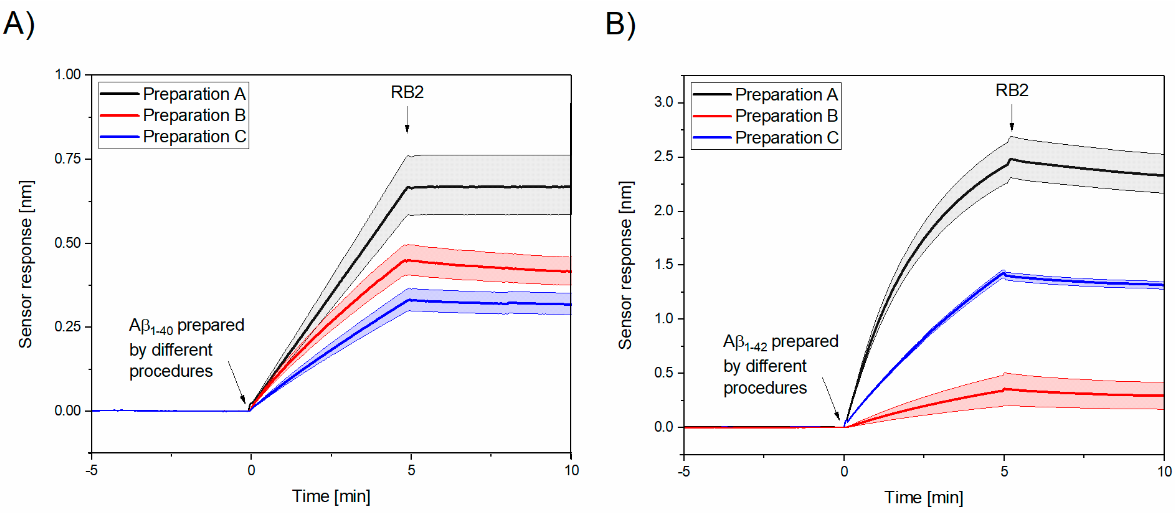

Figure S3: The effect of preparation of Aβ on its the binding to cypD. Typical sensor responses to the binding of A) $A\beta_{1-40}$ and B) $A\beta_{1-42}$ prepared by Preparation A-C to the immobilized cypD with marked standard deviations calculated from at least three measurements.

Comparison of interaction properties of 17β-HSD10$_{comercial}$ and 17β-HSD10$_{UHK}$

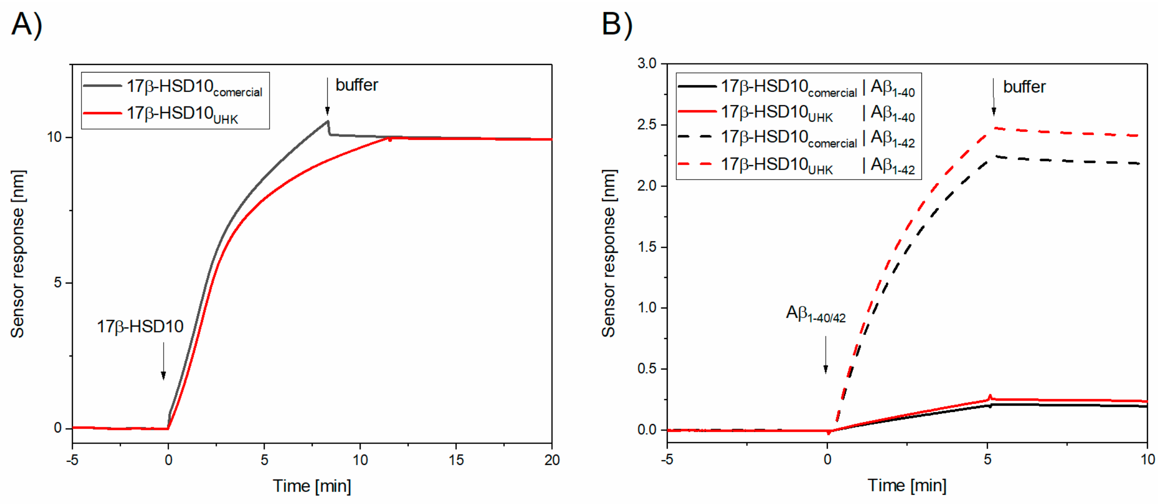

Figure S4: Comparison of the binding properties of commercially available 17β-HSD10 (17β-HSD10$_{comercial}$) and 17β-HSD10 with verified catalytic activity (17β-HSD10$_{UHK}$). A) Sensor response to the binding of 17β-HSD10 to the SPR surface with immobilized Ab(17β-HSD10), B) Sensor response to the binding of $A\beta_{1-40}$ and $A\beta_{1-42}$ to the captured 17β-HSD10.

Modelling the complex biomolecular interaction interplay in mitochondria

The model developed within our work assumes a system involving four concurrent first-order interactions (between Aβ_{1-40} or Aβ_{1-42} and cypD or 17β-HSD10) represented by Equation 1 in the main text. It describes the time evolution of this system for given initial conditions by calculating the rate of the concurrent reactions and determining the temporal concentrations of the interacting molecules and their complexes.

For a general reaction:

$$A + B \leftrightharpoons^{k_a}_{k_d} A/B$$

the reaction rate follows the first-order kinetics that may be expressed by the following equation:

$$\frac{d([AB])}{dt} = k_a \times [A] \times [B] - k_d \times [A/B]$$

In this equation, t is an independent time variable; [A], [B] and [A/B] denote the temporal concentrations of A, B and AB, respectively; and k_a and k_d denote the association and dissociation rate constants, respectively. The time-dependent concentrations of the interacting biomolecules in Equation 1 in the main text may be expressed as follows:

$$[cypD] = [cypD]_{t=0} - [A\beta_{1-40}/cypD] - [A\beta_{1-42}/cypD]$$

$$[17\beta - HSD10] = [17\beta - HSD10]_{t=0} - [A\beta_{1-40}/17\beta - HSD10] - [A\beta_{1-42}/17\beta - HSD10]$$

$$[A\beta_{1-40}] = [A\beta_{1-40}]_{t=0} - [A\beta_{1-40}/cypD] - [A\beta_{1-40}/17\beta - HSD10]$$

$$[A\beta_{1-42}] = [A\beta_{1-42}]_{t=0} - [A\beta_{1-42}/cypD] - [A\beta_{1-42}/17\beta - HSD10]$$

By inserting these expressions into the rate equation, a set of four differential equations was obtained:

$$\frac{d([A\beta_{1-40}/cypD])}{dt} = k_{a1} \times ([cypD]_{t=0} - [A\beta_{1-40}/cypD] - [A\beta_{1-42}/cypD]) \times ([A\beta_{1-40}]_{t=0} - [A\beta_{1-40}/cypD] - [A\beta_{1-40}/17\beta - HSD10]) - k_{d1} \times [A\beta_{1-40}/cypD]$$

$$\frac{d([A\beta_{1-42}/cypD])}{dt} = k_{a2} \times ([cypD]_{t=0} - [A\beta_{1-40}/cypD] - [A\beta_{1-42}/cypD]) \times ([A\beta_{1-42}]_{t=0} - [A\beta_{1-42}/cypD] - [A\beta_{1-42}/17\beta - HSD10]) - k_{d2} \times [A\beta_{1-42}/cypD]$$

$$\frac{d([A\beta_{1-40}/\ 17\beta - HSD10])}{dt} = k_{a3} \times ([17\beta - HSD10]_{t=0} - [A\beta_{1-40}/17\beta - HSD10]$$

$$- [A\beta_{1-42}/17\beta - HSD10]) \times ([A\beta_{1-40}]_{t=0} - [A\beta_{1-40}/cypD] - [A\beta_{1-40}/17\beta - HSD10])$$

$$- k_{d3} \times [A\beta_{1-40}/17\beta - HSD10]$$

$$\frac{d([A\beta_{1-42}/\ 17\beta - HSD10])}{dt} = k_{a4} \times ([17\beta - HSD10]_{t=0} - [A\beta_{1-40}/17\beta - HSD10]$$

$$- [A\beta_{1-42}/17\beta - HSD10]) \times ([A\beta_{1-42}]_{t=0} - [A\beta_{1-42}/cypD] - [A\beta_{1-42}/17\beta - HSD10])$$

$$- k_{d4} \times [A\beta_{1-42}/17\beta - HSD10]$$

Solving of these differential equations requires a set of accompanying initial conditions. In our simulations, we used the parameters listed in Table S1. The set of differential equations were solved using MATLAB 2016 software providing the temporal concentrations of individual biomolecules and their complexes as depicted in Figure 3 in the main text.

Table S1: Parameters of the multi-interaction model for interactions between Aβ, cypD and 17β-HSD10 under selected conditions associated with the progression of AD. All concentrations are given in molar units. The used k_a, k_d are listed in Table 1 and Table 2 in the main text.

	Physiological state	AD1	AD2	AD3
**Description of the situation**	$A\beta_{1-40}$:$A\beta_{1-42}$ ~ 9:1; low [Aβ]; Aβ monomeric	Preformed complexes; $A\beta_{1-40}$:$A\beta_{1-42}$ ~ 1:1; low [Aβ]; Aβ monomeric	Preformed complexes; $A\beta_{1-40}$:$A\beta_{1-42}$ ~ 1:1; high [Aβ]; Aβ monomeric	Preformed complexes; $A\beta_{1-40}$:$A\beta_{1-42}$ ~ 1:1; high [Aβ]; $A\beta_{1-42}$ oligomeric
**$[cypD]_{t=0}$**	1×10^{-7}	1×10^{-7}	1×10^{-7}	1×10^{-7}
**$[17\beta\text{-HSD10}]_{t=0}$**	7×10^{-7}	7×10^{-7}	7×10^{-7}	7×10^{-7}
**$[A\beta_{1-40}]_{t=0}$**	9×10^{-12}	5×10^{-12}	9×10^{-10}	5×10^{-10}
**$[A\beta_{1-42}]_{t=0}$**	1×10^{-12}	5×10^{-12}	1×10^{-10}	5×10^{-10}
**$[A\beta_{1-40}/\ cypD]_{t=0}$**	0	5.5×10^{-14}	5.5×10^{-14}	5.5×10^{-14}
**$[A\beta_{1-42}/\ cypD]_{t=0}$**	0	2.8×10^{-13}	2.8×10^{-13}	2.8×10^{-13}
**$[A\beta_{1-40}/17\beta\text{-HSD10}]_{t=0}$**	0	7.6×10^{-12}	7.6×10^{-12}	7.6×10^{-12}
**$[A\beta_{1-42}/17\beta\text{-HSD10}]_{t=0}$**	0	5.8×10^{-13}	5.8×10^{-13}	5.8×10^{-13}
**k_a, k_d for interactions with $A\beta_{1-40}$**	Table 1	Table 1	Table 1	Table 1
**k_a, k_d for interactions with $A\beta_{1-42}$**	Table 1	Table 1	Table 1	Table 2
